# Diagnostic Performance of Post-Treatment F-18 FDG PET/CT for Detecting Recurrent Cervical Cancer

**DOI:** 10.3390/diagnostics16172691

**Published:** 2026-08-23

**Authors:** Choon-Young Kim, Sang-Woo Lee, Gun Oh Chong, Jong Mi Kim, Yoon Hee Lee, Chae Moon Hong, Shin Young Jeong

**Affiliations:** 1 Department of Nuclear Medicine, Kyungpook National University Hospital, School of Medicine, Kyungpook National University, Daegu 41944, Republic of Korea; omh4ever@gmail.com (C.-Y.K.);; 2 Department of Nuclear Medicine, Kyungpook National University Chilgok Hospital, School of Medicine, Kyungpook National University, Daegu 41404, Republic of Korea; 3 Department of Obstetrics and Gynecology, Kyungpook National University Chilgok Hospital, School of Medicine, Kyungpook National University, Daegu 41404, Republic of Korea

**Keywords:** cervical cancer, recurrence, follow-up imaging, F-18 FDG PET/CT

## Abstract

**Background/Objectives:** Cervical cancer recurrence after definitive treatment remains a major clinical challenge, and early detection may improve patient outcomes. This study evaluated the diagnostic performance of fluorine-18 fluorodeoxyglucose (F-18 FDG) positron emission tomography/computed tomography (PET/CT) for detecting recurrent cervical cancer according to clinical context. **Methods:** We retrospectively reviewed 1727 post-treatment PET/CT examinations obtained from 659 patients who achieved a complete response after definitive treatment between 2005 and 2024. After excluding 15 examinations that detected second primary malignancies, the recurrence-specific analysis included 1712 examinations from 654 patients. PET/CT findings were classified as true positive, true negative, false positive, or false negative based on histopathology or serial imaging follow-up. Generalized estimating equations were used to account for within-patient clustering. Subgroup analyses were performed according to histology, International Federation of Gynecology and Obstetrics (FIGO) 2009 stage, interval from treatment completion to PET/CT, and clinical context. **Results:** Of the 1712 examinations, 136 (7.9%) were true positive, 1525 (89.1%) were true negative, 43 (2.5%) were false positive, and 8 (0.5%) were false negative. Overall sensitivity, specificity, positive predictive value (PPV), negative predictive value (NPV), and accuracy were 94.4%, 97.3%, 76.0%, 99.5%, and 97.0%, respectively. Diagnostic performance was similar across histologic subtypes and FIGO stages. In routine follow-up settings without clinical suspicion of recurrence, PET/CT showed a high NPV (100.0%), whereas examinations performed for clinically suspected recurrence showed a high PPV (96.5%). **Conclusions:** These findings suggest that the diagnostic interpretation and clinical utility of post-treatment PET/CT may vary according to the clinical setting.

## 1. Introduction

Cervical cancer is the fourth most common cancer among women worldwide [1]. In the Republic of Korea, cervical cancer was the 11th most common cancer among women in 2023, with 3144 newly diagnosed cases nationwide [2]. Although cervical cancer incidence has declined with the implementation of organized screening programs and is expected to decrease further with expanded human papillomavirus vaccination [3,4], recurrence remains a major clinical challenge, particularly in patients with advanced-stage disease. Approximately one-third of patients with advanced-stage disease eventually develop recurrent cervical cancer after definitive treatment, most commonly within the first two years [5]. Prognosis after recurrence is generally poor; however, early detection and timely management may improve outcomes in selected patients [6].

Post-treatment follow-up strategies traditionally rely on symptom assessment, physical examination, serum tumor markers such as squamous cell carcinoma antigen (SCC-Ag) and carcinoembryonic antigen (CEA), Papanicolaou (Pap) cytology, and conventional imaging modalities [7,8]. However, serum tumor marker elevation does not always correlate with recurrent disease [9], and conventional imaging may have limited sensitivity for detecting small-volume or clinically occult lesions [10]. Fluorine-18 fluorodeoxyglucose (F-18 FDG) positron emission tomography/computed tomography (PET/CT) has emerged as a useful imaging modality because metabolic abnormalities may precede structural changes detectable on conventional imaging [11].

Beyond the initial post-treatment assessment, current guidelines do not recommend routine serial PET/CT surveillance in asymptomatic patients after treatment for cervical cancer. The 2026 Society of Gynecologic Oncology clinical practice statement and the National Comprehensive Cancer Network (NCCN) guidelines generally reserve additional PET/CT imaging for patients with symptoms, abnormal examination findings, or other clinical evidence suggestive of recurrence [7,8]. This limited role in routine surveillance reflects the absence of established evidence for a clinical benefit from routine imaging and uncertainty regarding its cost-effectiveness [8,12,13,14]. Nevertheless, surveillance PET/CT has demonstrated high diagnostic performance in several malignancies, with reported sensitivities and specificities of 98.9% and 98.1% in non-small cell lung cancer [15], 97.9% and 98.9% in head and neck squamous cell carcinoma [16], 100% and 94.0% in esophageal cancer [17], and 100% and 98.5% in breast cancer [18], respectively.

Previous studies have reported favorable diagnostic performance of post-treatment FDG PET or PET/CT for detecting recurrent cervical cancer, with sensitivities ranging from 85.7% to 100.0% and specificities ranging from 83.3% to 87.8% [19,20,21,22]. Brooks et al. specifically investigated serial surveillance FDG PET in 103 patients who had achieved a complete metabolic response after definitive chemoradiotherapy and demonstrated that surveillance PET could detect asymptomatic recurrence [5]. However, previous studies generally included relatively small or selected populations, with imaging contexts ranging from routine surveillance of asymptomatic patients to evaluation of unexplained tumor marker elevation or clinically suspected recurrence. Moreover, they did not provide comprehensive diagnostic performance estimates stratified by clinical indication. Because the pretest probability of recurrence may differ substantially according to the indication for imaging, the diagnostic characteristics and potential clinical utility of PET/CT should be evaluated according to the clinical context in which the examination is performed.

Therefore, this study aimed to evaluate the diagnostic performance of post-treatment PET/CT for detecting recurrent cervical cancer after definitive treatment using a large real-world cohort, with particular emphasis on its performance in routine follow-up and clinically suspected recurrence settings.

## 2. Materials and Methods

### 2.1. Patients and Study Design

This retrospective study was conducted to evaluate the diagnostic performance of post-treatment PET/CT for detecting recurrent cervical cancer after definitive treatment. All procedures performed in this study involving human participants were in accordance with the ethical standards of the institutional research committee and with the Declaration of Helsinki and its later amendments.

We reviewed 822 consecutive patients who were diagnosed with cervical cancer, completed definitive treatment, achieved complete response, and underwent at least one PET/CT examination at our institution between January 2005 and December 2024. Clinical information, including age, histopathologic subtype, International Federation of Gynecology and Obstetrics (FIGO) stage, treatment modality, treatment response, follow-up duration, and recurrence status, was obtained from electronic medical records.

Cervical cancer staging was assigned according to the 2009 FIGO staging system [23]. Definitive treatment consisted of surgery alone, surgery followed by adjuvant therapy, or definitive concurrent chemoradiotherapy (CCRT), depending on disease stage and clinical presentation. Neoadjuvant therapy was administered when clinically indicated. A complete response was determined based on clinical examination, conventional imaging, and laboratory evaluation according to institutional follow-up protocols.

Patients were excluded for insufficient clinical or imaging data (missing information or loss to follow-up; *n* = 51), carcinoma in situ (*n* = 34), FIGO stage IVB disease at diagnosis (*n* = 17), or another concurrent primary malignancy at the time of cervical cancer diagnosis or treatment (*n* = 15). After exclusion, 705 patients remained eligible for analysis. All PET/CT examinations were reviewed to determine the clinical indication at the time of imaging.

**Figure 1:**
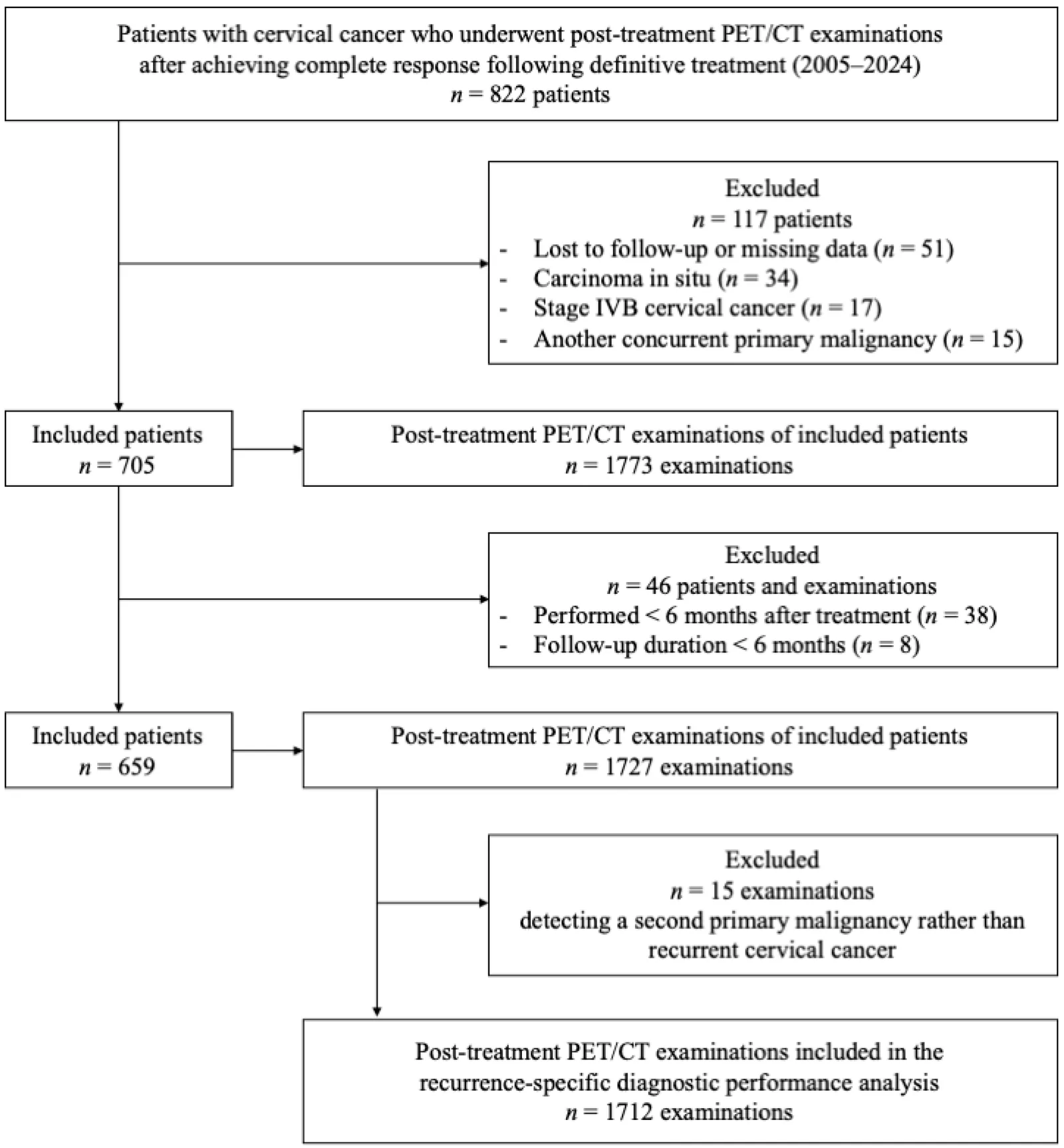
Flow diagram of patient selection and inclusion of post-treatment PET/CT examinations.

PET/CT examinations performed during post-treatment follow-up were evaluated regardless of whether they were obtained as part of routine follow-up or because recurrence was clinically suspected. Among 1773 post-treatment PET/CT examinations obtained from the 705 eligible patients, 38 examinations performed within 6 months after treatment completion were excluded to minimize the inclusion of persistent or residual disease [24]. An additional eight examinations with negative PET/CT findings were excluded because less than 6 months of subsequent clinical or imaging follow-up was available; a minimum follow-up duration of 6 months was required to classify a negative PET/CT finding as true negative [21,25]. Finally, 659 patients and 1727 post-treatment PET/CT scans were included in the analyses. A flow diagram of patient selection is shown in Figure 1.

### 2.2. Post-Treatment Follow-Up Strategy

Post-treatment follow-up was generally performed in accordance with Society of Gynecologic Oncology recommendations [12]. Clinical evaluations were generally scheduled approximately every 3 months during the first 2 years after treatment, every 6 months during the subsequent 3 years, and annually thereafter. Follow-up evaluations included symptom assessment, physical examination, Pap cytology when appropriate, and serum tumor marker measurements, including SCC-Ag and CEA. Conventional imaging was performed according to institutional practice and clinical indications and generally included abdominopelvic computed tomography (CT) or magnetic resonance imaging (MRI) and chest radiography.

PET/CT was performed either as part of routine post-treatment follow-up or when recurrence was clinically suspected because of new or worsening symptoms, abnormal physical examination findings, elevated serum tumor markers, abnormal cytologic or histopathologic findings, or indeterminate findings on conventional imaging. Because PET/CT is used in both routine follow-up and clinically suspected recurrence settings in real-world practice, examinations performed in both settings were included. PET/CT was not mandated at uniform intervals for all patients who achieved a complete response, and the decision to perform PET/CT reflected institutional practice and physician judgment.

For subgroup analysis, each PET/CT examination was categorized according to the clinical context at the time of imaging: (1) routine follow-up, defined as an examination performed in the absence of clinical evidence suggestive of recurrence; or (2) clinically suspected recurrence, defined as an examination performed in the presence of one or more of the following: new or worsening symptoms, abnormal physical examination findings, elevated serum tumor markers, abnormal cytologic or histopathologic findings, or indeterminate findings on conventional imaging.

### 2.3. Image Acquisition and Interpretation

F-18 FDG PET/CT scans were acquired using either a Reveal RT-HiREZ PET/CT scanner equipped with a 6-slice CT component (CTI Molecular Imaging, Knoxville, TN, USA) or a Discovery STe PET/CT scanner equipped with a 16-slice CT component (GE Healthcare, Milwaukee, WI, USA), according to a previously described institutional protocol [26]. All patients fasted for at least 6 h before F-18 FDG administration, and blood glucose levels were measured before tracer injection. Patients with blood glucose levels exceeding 150 mg/dL had their examinations rescheduled or underwent appropriate glycemic control before imaging. F-18 FDG was administered intravenously at a dose of approximately 3.7–5.55 MBq/kg, and imaging was initiated approximately 60 min after tracer administration. Low-dose non-contrast CT images were initially obtained from the skull base to the proximal thighs for anatomical localization and attenuation correction, with the patients in the supine position and breathing quietly. PET emission images were subsequently acquired over the same anatomical range at 3 min per bed position. The maximum spatial resolutions were 6.5 mm for the Reveal system and 5.5 mm for the Discovery system. PET images were reconstructed using a 128 × 128 matrix and an ordered-subset expectation maximization algorithm with four iterations and eight subsets, followed by application of a 5.0 mm Gaussian filter. The reconstructed slice thickness was 3.0 mm for the Reveal system and 3.27 mm for the Discovery system [26].

All PET/CT images were independently interpreted by two experienced nuclear medicine physicians who were aware that the patients had previously undergone treatment for cervical cancer. Image interpretation was based on visual assessment supported by semi-quantitative analysis and comparison with prior imaging studies. Focal FDG uptake inconsistent with physiologic activity or normal biodistribution was considered suspicious for recurrence. Discrepancies between readers were resolved by consensus review. Incidental FDG uptake suggestive of a malignancy unrelated to cervical cancer was separately categorized as a possible second primary malignancy.

### 2.4. Diagnostic Performance Assessment

Each PET/CT examination was classified as true positive, false positive, true negative, or false negative for recurrent cervical cancer. A positive PET/CT finding was classified as true positive when recurrence was confirmed histopathologically. When histopathologic confirmation was unavailable, recurrence was determined based on serial imaging findings, clinical follow-up, and multidisciplinary assessment. Radiologic confirmation of recurrence required the appearance of a new lesion or progressive enlargement or increasing metabolic activity of a suspected lesion on follow-up imaging. A positive PET/CT finding was classified as false positive when histopathologic evaluation demonstrated a benign lesion or subsequent CT, MRI, or other follow-up imaging showed no evidence of recurrence. A negative PET/CT finding was classified as true negative when no recurrence was identified during at least 6 months of subsequent clinical and imaging follow-up and as false negative when recurrence was identified histopathologically despite the negative PET/CT finding.

### 2.5. Statistical Analysis

Continuous variables are presented as mean ± standard deviation, and categorical variables are presented as frequencies and percentages. Subgroup analyses were performed according to histologic subtype, FIGO stage, interval from treatment completion to PET/CT, and clinical context at the time of imaging. Diagnostic performance metrics, including sensitivity, specificity, positive predictive value (PPV), negative predictive value (NPV), and accuracy, with corresponding 95% confidence intervals (CIs), were calculated. Because multiple PET/CT examinations were performed in the same patients, generalized estimating equations (GEE) with robust standard errors were used to account for within-patient clustering in the scan-level analyses [27]. *P*-values were obtained using two-sided Wald tests from the corresponding GEE models. When GEE estimates could not be obtained because of a lack of outcome variation, exact 95% CIs were calculated using the Clopper–Pearson method. All statistical tests were two-sided, and *p*-values < 0.05 were considered statistically significant. Statistical analyses were performed using IBM SPSS Statistics version 31.0 (IBM Corp., Armonk, NY, USA).

## 3. Results

### 3.1. Characteristics of the Study Population

A total of 659 patients with 1727 post-treatment PET/CT examinations were included in the study cohort. Baseline characteristics of the study population are summarized in Table 1. The mean patient age was 51.2 ± 12.1 years. Squamous cell carcinoma (SqCC) was the predominant histologic subtype (*n* = 520, 78.9%). The most common FIGO stage was stage IB (*n* = 278, 42.2%), followed by stage IIB (*n* = 214, 32.5%).

Regarding primary treatment, 257 patients (39.0%) underwent surgery alone, whereas 191 (29.0%) received definitive CCRT. Overall, 211 patients (32.0%) underwent a single post-treatment PET/CT examination, whereas 90 (13.7%) underwent five or more examinations.

### 3.2. Patterns and Confirmation of Post-Treatment PET/CT Findings

Post-treatment PET/CT detected 15 second primary malignancies: lung cancer (*n* = 8), thyroid cancer (*n* = 1), tongue cancer (*n* = 1), colon cancer (*n* = 2), vulvar cancer (*n* = 1), a spindle cell neoplasm of the abdominal wall (*n* = 1), and pancreatic cancer (*n* = 1). After excluding the 15 examinations that detected second primary malignancies, the recurrence-specific diagnostic performance analysis included 1712 examinations from 654 patients.

Among these examinations, 136 (7.9%) were true positive, 1525 (89.1%) were true negative, 43 (2.5%) were false positive, and 8 (0.5%) were false negative. Multiple-organ metastases (*n* = 44) and distant lymph node metastases (*n* = 30) were the most common recurrence patterns among the true-positive examinations. All eight false-negative findings represented central pelvic recurrence (Table 2).

Among the 136 true-positive examinations, recurrent cervical cancer was confirmed histopathologically in 66 (48.5%) and by serial imaging follow-up, supported by multidisciplinary assessment when required, in 70 (51.5%). Among the 43 false-positive examinations, 18 (41.9%) were confirmed as benign by histopathologic evaluation, whereas 25 (58.1%) showed no evidence of recurrence on subsequent CT, MRI, or other follow-up imaging. All eight false-negative examinations were histopathologically confirmed as recurrent cervical cancer.

### 3.3. Distribution of Post-Treatment PET/CT Findings According to Subgroup

The distribution of true-positive, true-negative, false-positive, and false-negative PET/CT examinations according to histologic subtype, FIGO stage, interval from treatment completion to PET/CT, and clinical context is summarized in Table 3. Among examinations performed in patients with SqCC, 96 (7.1%) were classified as true positive and 1213 (89.9%) as true negative, whereas the corresponding values for non-SqCC histology were 40 (11.0%) and 312 (86.0%), respectively. The proportion of true-positive examinations was 12.4% among examinations from patients with FIGO stage II–IVA disease and 4.8% in those with stage I disease. When stratified according to the interval from treatment completion to PET/CT, the proportion of true-positive examinations was 11.4% within 2 years after treatment and 5.2% more than 2 years after treatment. True-positive examinations accounted for 44.1% of examinations performed for clinically suspected recurrence and 3.5% of routine follow-up examinations, whereas true-negative examinations accounted for 93.8% of routine follow-up examinations.

### 3.4. Diagnostic Performance of Post-Treatment PET/CT for Detecting Recurrent Cervical Cancer

After accounting for within-patient clustering using GEE, post-treatment PET/CT demonstrated an overall sensitivity of 94.4%, specificity of 97.3%, PPV of 76.0%, NPV of 99.5%, and accuracy of 97.0% (Table 4). No significant differences in diagnostic performance were observed between examinations performed in patients with SqCC and those performed in patients with non-SqCC histology. Sensitivity was 96.0% in the SqCC group and 90.9% in the non-SqCC group (*p* = 0.218), and specificity was similarly high in both groups (97.1% vs. 97.8%, *p* = 0.539). PPV (72.7% vs. 85.1%, *p* = 0.108), NPV (99.7% vs. 98.7%, *p* = 0.055), and accuracy (97.0% in both groups, *p* = 0.950) also did not differ significantly.

Diagnostic performance did not differ significantly between patients with FIGO stage I disease and those with stage II–IVA disease. Sensitivity was 90.6% versus 96.7% (*p* = 0.129), and specificity was 97.8% versus 96.5% (*p* = 0.133), respectively. PPV (69.6% vs. 80.0%, *p* = 0.120), NPV (99.5% in both groups, *p* = 0.918), and accuracy (97.4% vs. 96.5%, *p* = 0.285) were also comparable.

When stratified according to the interval between treatment completion and PET/CT, sensitivity, PPV, and NPV were similar between the ≤2 years and >2 years groups. However, specificity (95.5% vs. 98.6%, *p* < 0.001) and accuracy (95.6% vs. 98.1%, *p* = 0.002) were significantly higher in the >2 years group (Table 4).

### 3.5. Diagnostic Performance of Post-Treatment PET/CT According to Clinical Context

Among the 1,712 post-treatment PET/CT examinations, 1,526 (89.1%) were categorized as routine follow-up examinations and 186 (10.9%) as examinations performed for clinically suspected recurrence (Table 3). Each examination in the clinically suspected recurrence group was assigned to a single category according to the primary indication for PET/CT: isolated tumor marker elevation (*n* = 117), indeterminate findings on conventional imaging (*n* = 42), new or worsening symptoms and/or abnormal physical examination findings (*n* = 12), or abnormal Pap cytology or biopsy results (*n* = 15). Examinations in the latter two categories were classified as such irrespective of concomitant tumor marker elevation.

In the routine follow-up group, PET/CT demonstrated a sensitivity of 100.0%, specificity of 97.3%, PPV of 57.4%, NPV of 100.0%, and accuracy of 97.4%. In the clinically suspected recurrence group, the corresponding values were 91.1%, 96.9%, 96.5%, 92.1%, and 94.1%, respectively (Table 4). The prevalence of recurrence was 3.5% in routine follow-up examinations and 48.4% in examinations performed for clinically suspected recurrence. These distinct PPV and NPV profiles are consistent with the substantial difference in the pretest probability of recurrence between the two clinical contexts.

## 4. Discussion

In this large retrospective cohort study, post-treatment PET/CT demonstrated high diagnostic performance for detecting recurrent cervical cancer after definitive treatment, with high sensitivity, specificity, NPV, and overall accuracy, although the overall PPV was moderate (76.0%), largely reflecting the low prevalence of recurrence in routine follow-up examinations. In addition to recurrent cervical cancer, PET/CT identified 15 unexpected second primary malignancies, suggesting an additional potential clinical benefit of PET/CT during post-treatment follow-up. Chen et al. suggested that cervical cancer survivors, particularly those treated with radiotherapy, are at increased risk of second primary malignancies [28].

The patterns of false-positive and false-negative findings provide insight into the strengths and limitations of PET/CT in this clinical setting. False-positive findings most commonly involved pelvic or distant lymph nodes and may have reflected inflammatory or reactive changes, which are well-recognized limitations of FDG PET/CT [24,29]. In contrast, all false-negative cases were biopsy-proven central pelvic recurrences. Small-volume disease below the effective spatial resolution of PET/CT may contribute to false-negative findings [30]. These findings highlight the importance of integrating PET/CT findings with clinical assessment and other imaging modalities during post-treatment follow-up.

The diagnostic performance of post-treatment PET/CT remained consistently high regardless of histologic subtype or FIGO stage, indicating that its diagnostic reliability was maintained across diverse disease characteristics. Although current NCCN guidelines mainly emphasize PET/CT within the early post-treatment period, particularly for patients with locally advanced disease [7], diagnostic performance remained high beyond two years in this cohort, while the clinical value and optimal timing of late surveillance PET/CT require prospective evaluation.

A major finding of this study is that PET/CT showed distinct diagnostic performance profiles according to the clinical context in which the examination was performed. In routine follow-up examinations without clinical suspicion of recurrence, PET/CT demonstrated a very high NPV. However, the absence of false-negative findings in this subgroup should be interpreted cautiously given the relatively low prevalence of recurrence. In examinations performed for clinically suspected recurrence, PET/CT demonstrated a high PPV, likely reflecting the higher pretest probability of recurrent disease in this setting. These findings suggest that PET/CT may serve different but complementary clinical roles depending on the pretest probability of recurrence.

Previous studies evaluating post-treatment FDG PET or PET/CT for recurrent cervical cancer differed considerably in patient selection and imaging indication. Havrilesky et al. reported a sensitivity of 85.7% and a specificity of 86.7% based on 29 FDG PET examinations in 22 patients with clinically suspected recurrence [19]. Chong et al. evaluated 32 patients with SqCC and unexplained serial elevation of serum tumor markers and reported a sensitivity of 100.0% and a specificity of 83.3% [20]. Chung et al. evaluated 121 patients who had achieved a complete response after primary treatment, including both symptomatic and asymptomatic patients, and reported a sensitivity of 96.1% and a specificity of 84.4% [21]. In a subsequent study involving 276 patients undergoing post-treatment PET/CT, Chung et al. reported corresponding values of 94.7% and 87.8%, respectively [22]. Thus, the diagnostic performance observed in the present study was broadly consistent with previous reports while extending the available evidence to a substantially larger real-world cohort that included serial PET/CT examinations and separate analyses according to clinical context.

Taken together, our findings suggest that post-treatment PET/CT may be most useful when interpreted according to clinical context rather than applied uniformly across all follow-up settings. In routine follow-up settings, the high NPV should be interpreted in the context of the low prevalence of recurrence and does not, by itself, establish the clinical benefit of routine PET/CT surveillance. In clinically suspected recurrence settings, the high PPV may facilitate prompt clinical decision-making and treatment planning. Routine PET/CT surveillance remains controversial because its clinical benefit has not been established and its cost-effectiveness remains uncertain [8,12,13,14]. Although cost-effectiveness was not evaluated in this study, the context-specific diagnostic performance observed in our cohort may help inform more individualized follow-up strategies.

This study has several strengths. First, it included a large number of patients and PET/CT examinations collected over an extended study period. Second, both routine follow-up and clinically suspected recurrence settings were evaluated, allowing PET/CT performance to be characterized in relation to differences in pretest probability. Third, recurrence status was determined using histopathologic confirmation or serial imaging follow-up, providing a clinically relevant reference standard.

Several limitations should be acknowledged. First, this was a retrospective single-center study, which may limit generalizability and introduce selection bias regarding the indication and frequency of PET/CT examinations. In addition, not all lesions were pathologically confirmed, and some recurrence determinations relied on serial imaging follow-up and multidisciplinary assessment, potentially introducing verification bias. Variations in treatment modality, follow-up interval, and physician decision-making may also have influenced PET/CT utilization and diagnostic performance. Although the same two PET/CT systems and corresponding acquisition and reconstruction protocols were used throughout the study period, the extended period from 2005 to 2024 may have introduced temporal heterogeneity related to changes in cervical cancer treatment strategies, follow-up practices, and guideline recommendations. Furthermore, although the PET/CT examinations were interpreted independently by two nuclear medicine physicians and disagreements were resolved by consensus, interobserver agreement was not quantitatively assessed. Finally, the primary analysis was performed on a per-scan basis, and individual patients contributed different numbers of examinations. Generalized estimating equations with robust standard errors were used to account for within-patient clustering; nevertheless, the findings may have been influenced by unequal examination frequency and the selective use of repeated PET/CT in patients with different risks of recurrence. Prospective studies evaluating clinical outcomes, cost-effectiveness, and optimal imaging schedules are needed before routine PET/CT surveillance can be recommended.

## 5. Conclusions

Post-treatment PET/CT demonstrated high diagnostic performance for detecting recurrent cervical cancer in this retrospective cohort. The diagnostic characteristics and potential clinical utility of PET/CT differed according to the clinical context in which the examination was performed. In patients undergoing routine follow-up without clinical suspicion of recurrence, PET/CT demonstrated a very high NPV, whereas in clinically suspected recurrence settings, positive PET/CT findings were highly associated with true recurrence. These findings support context-specific interpretation of PET/CT findings during post-treatment follow-up of cervical cancer.

## Figures and Tables

**Table 1:** Demographic and clinical characteristics of patients (*n* = 659).

Characteristics	No. of Patients (%)
Age at diagnosis, years <40 40–50 51–60 >60	118 (17.9%) 212 (32.2%) 181 (27.5%) 148 (22.5%)
Histology Squamous cell carcinoma Adenocarcinoma Other	520 (78.9%) 126 (19.1%) 13 (2.0%)
FIGO stage Stage IA Stage IB Stage IIA Stage IIB Stage IIIA Stage IIIB Stage IVA	94 (14.3%) 278 (42.2%) 44 (6.7%) 214 (32.5%) 12 (1.8%) 10 (1.5%) 7 (1.1%)
Primary treatment modality * Surgery only Surgery + adjuvant chemotherapy Surgery + adjuvant radiotherapy Surgery + adjuvant CCRT Definitive CCRT	257 (39.0%) 71 (10.8%) 34 (5.2%) 106 (16.1%) 191 (29.0%)
Number of post-treatment PET/CT scans for each patient 1 2 3 4 ≥5	211 (32.0%) 133 (20.2%) 136 (20.6%) 89 (13.5%) 90 (13.7%)

CCRT, concurrent chemoradiotherapy. * Neoadjuvant therapy was administered when clinically indicated.

**Table 2:** Anatomic distribution of true-positive, false-positive, and false-negative PET/CT findings for recurrent cervical cancer.

		True Positive (*n* = 136, 7.9%)	False Positive (*n* = 43, 2.5%)	False Negative (*n* = 8, 0.5%)
Locoregional recurrence	Central pelvic recurrence *	23 (16.9%)	7 (16.3%)	8 (100.0%)
	Lateral pelvic recurrence **^†^**	18 (13.2%)	13 (30.2%)	0 (0.0%)
Distant metastasis	Distant lymph node	30 (22.1%)	15 (34.9%)	0 (0.0%)
	Lung	15 (11.0%)	1 (2.3%)	0 (0.0%)
	Peritoneum	3 (2.2%)	4 (9.3%)	0 (0.0%)
	Other sites ^‡^	3 (2.2%)	1 (2.3%)	0 (0.0%)
	Multiple organ metastases	44 (32.4%)	2 (4.7%)	0 (0.0%)

All analyses were performed on a per-scan basis. Fifteen examinations detecting second primary malignancies were excluded from the recurrence-specific scan-level analysis, resulting in the inclusion of 1712 examinations from 654 patients. * Central pelvic recurrence: vaginal stump, uterine cervix, vagina, central parametrium, and the adjacent pelvic spaces. ^†^ Lateral pelvic recurrence: pelvic sidewall, pelvic lymph nodes (obturator, internal iliac, external iliac, common iliac, and presacral lymph nodes). ^‡^ Other sites: kidney (true positive), bone (true positive), abdominal wall (true positive), and rectum (false positive).

**Table 3:** Classification of PET/CT examinations in the recurrence-specific scan-level analysis according to subgroup.

		No. of Scans	No. of Patients *	True positive	True Negative	False Positive	False Negative
Total		1712	654	136 (7.9%)	1525 (89.1%)	43 (2.5%)	8 (0.5%)
Histology	SqCC Non-SqCC	1349 (78.8%) 363 (21.2%)	515 139	96 (7.1%) 40 (11.0%)	1213 (89.9%) 312 (86.0%)	36 (2.7%) 7 (1.9%)	4 (0.3%) 4 (1.1%)
FIGO stage	I II–IVA	1000 (58.4%) 712 (41.6%)	371 283	48 (4.8%) 88 (12.4%)	926 (92.6%) 599 (84.1%)	21 (2.1%) 22 (3.1%)	5 (0.5%) 3 (0.4%)
Interval from treatment completion to PET/CT	≤2 years >2 years	754 (44.0%) 958 (56.0%)	475 449	86 (11.4%) 50 (5.2%)	635 (84.2%) 890 (92.9%)	30 (4.0%) 13 (1.4%)	3 (0.4%) 5 (0.5%)
Routine follow-up		1526 (89.1%)	589	54 (3.5%)	1432 (93.8%)	40 (2.6%)	0 (0.0%)
Clinically suspected recurrence		186 (10.9%)	148	82 (44.1%)	93 (50.0%)	3 (1.6%)	8 (4.3%)

SqCC, squamous cell carcinoma; FIGO, International Federation of Gynecology and Obstetrics. Fifteen examinations detecting second primary malignancies were excluded, resulting in 1712 examinations from 654 patients for the recurrence-specific analysis. Data are presented as number (%) unless otherwise indicated. Percentages were calculated using the number of scans in each row as the denominator. * The number of patients represents the number of unique patients contributing at least one examination to each subgroup. Individual patients could contribute examinations to more than one interval or clinical-context category; therefore, patient counts across these subgroups do not necessarily sum to the total number of patients.

**Table 4:** Diagnostic performance of post-treatment PET/CT for detecting recurrent cervical cancer estimated using generalized estimating equations with patient-level clustering.

		Sensitivity	Specificity	PPV	NPV	Accuracy
All scans		94.4 (89.4–97.2)	97.3 (96.3–98.0)	76.0 (69.1–81.7)	99.5 (99.0–99.7)	97.0 (96.1–97.7)
Histology	SqCC	96.0 (89.9–98.5)	97.1 (96.0–97.9)	72.7 (64.5–79.6)	99.7 (99.1–99.9)	97.0 (96.0–97.8)
	Non-SqCC	90.9 (79.0–96.4)	97.8 (95.1–99.0)	85.1 (71.0–93.0)	98.7 (96.7–99.5)	97.0 (94.5–98.4)
	*p*-value	0.218	0.539	0.108	0.055	0.950
FIGO stage	I	90.6 (79.8–95.9)	97.8 (96.6–98.6)	69.6 (57.3–79.6)	99.5 (98.7–99.8)	97.4 (96.2–98.2)
	II–IVA	96.7 (90.4–98.9)	96.5 (94.6–97.7)	80.0 (71.6–86.4)	99.5 (98.5–99.8)	96.5 (94.8–97.6)
	P-value	0.129	0.133	0.120	0.918	0.285
Interval from treatment completion to PET/CT	≤2 years	96.6 (90.3–98.9)	95.5 (93.7–96.8)	74.1 (65.6–81.1)	99.5 (98.5–99.8)	95.6 (94.0–96.8)
	>2 years	90.9 (80.2–96.1)	98.6 (97.5–99.2)	79.4 (68.5–87.2)	99.4 (98.7–99.8)	98.1 (97.0–98.8)
	*p*-value	0.152	<0.001	0.385	0.814	0.002
Routine follow-up		100.0 (93.4–100.0) *	97.3 (96.3–98.0)	57.4 (47.2–67.1)	100.0 (99.7–100.0) *	97.4 (96.4–98.1)
Clinically suspected recurrence		91.1 (83.5–95.4)	96.9 (91.1–98.9)	96.5 (89.7–98.8)	92.1 (84.7–96.1)	94.1 (89.8–96.6)

PPV, positive predictive value; NPV, negative predictive value; SqCC, squamous cell carcinoma; FIGO, International Federation of Gynecology and Obstetrics; CI, confidence interval; GEE, generalized estimating equations. Values are percentages with 95% CIs. Estimates and CIs were obtained using GEE models with patient-level clustering and robust standard errors. *P*-values were obtained using two-sided Wald tests from the corresponding GEE models. * GEE estimates could not be obtained because of a lack of outcome variation (i.e., no false-negative examinations). The observed proportions and their 95% CIs calculated using the Clopper–Pearson exact method are presented.

## Data Availability

The data presented in this study are available on request from the corresponding author. The data are not publicly available due to institutional policy and patient privacy restrictions.
